# Intranasal Oxytocin as an adjunct treatment in patients with major depression with and without comorbid borderline personality disorder

**DOI:** 10.1192/j.eurpsy.2023.513

**Published:** 2023-07-19

**Authors:** H. Maoz

**Affiliations:** Shalvata mental health center, Tel-Aviv University, Hod Hasharon, Israel

## Abstract

**Introduction:**

The mechanisms leading to Oxytocin’s differential effects among patients with borderline personality disorder have thus far been elusive.

**Objectives:**

This study was aimed to explore the differential effect of OT administration among depressive patients with or without comorbid borderline personality disorder, and to explore the mediating role of attachment in these differential patterns.

**Methods:**

Patients treated with psychotherapy in an inpatient settings (N=58) were randomized and double-blindly allocated to receive oxytocin or placebo for a period of four weeks. The effect of OT on therapy process and outcome was examined among patients with (n=35) and without (n=23) borderline personality disorder. Moderated mediational models were estimated to explore whether attachment differentially affected the association between oxytocin and treatment outcomes.

**Results:**

patients without BPD showed significantly larger improvements following OT administration (B=-8.32, p=.001) as compared to placebo in OQ-45. On the other hand, patients with BPD showed no significant improvement following OT (B=0.61, p=.76). The same pattern was observed in the HSCL, where patients without BPD demonstrated significantly larger improvements following OT administration (B=-0.29 ,p=.0009) as compared to placebo, while patients with BPD demonstrated no significant improvement (B=-0.04 ,p=.55). Moderated mediational models indicated no significant moderated indirect effect, however, a significant trend of indirect effect only in the BPD group was observed, whereby the no-BPD group showed a stronger direct effect (β=-0.19, t=-1.30, p=.20), whereas the BPD group showed a stronger indirect effect (β=-0.72, SE=0.45, CI= -1,71, -0.00).

**Conclusions:**

Patients with depression and comorbid BPD benefit less from OT administration as compared depressive patients without such comorbidity. It is possible that the involvement of the attachment system may be associated with the attenuation of OT’s effect.

**Disclosure of Interest:**

None Declared

